# Arthroscopic Bankart repair versus conservative treatment for first-time traumatic anterior shoulder dislocation: a systematic review and meta-analysis

**DOI:** 10.1186/s40001-023-01160-0

**Published:** 2023-07-27

**Authors:** Bin Hu, Jianqiao Hong, Hanxiao Zhu, Shigui Yan, Haobo Wu

**Affiliations:** 1 Department of Orthopedic Surgery, The Second Affiliated Hospital, Zhejiang University School of Medicine, 1511# JiangHong Road, Hangzhou, 310009 China; 2 Orthopaedics Research Institute of Zhejiang University, Hangzhou, China

**Keywords:** Anterior shoulder dislocation, Arthroscopic Bankart repair, Meta-analysis, Recurrence, Return to play

## Abstract

**Background:**

Shoulder is vulnerable to dislocation owing to its anatomical structure and the increasing popularity of contact sports in young population. The management of first-time anterior shoulder dislocation in this group is still controversial and the prognosis are varied. This review aimed to compare the results of arthroscopic Bankart repair and conservative management for first-time traumatic anterior shoulder dislocation in young active patients.

**Methods:**

Databases were searched till November 2021, and comparative studies between arthroscopic Bankart repair and conservative management for first-time traumatic anterior shoulder dislocation in young population were selected. Methodological quality of the studies was assessed according to the Cochrane Back Review Group 12-item scale. Outcome measures included recurrence of instability, return to play, subsequent instability surgery, and shoulder functional scores.

**Results:**

The search returned 12 eligible trials with 786 participants. All the trials were of prospective design. After arthroscopic Bankart repair, patients experienced significantly less re-dislocation (7.5% vs. 53.0%, *p* < 0.00001, *I*^2^ = 0%), subluxation (3.1% vs. 24.2%, *p* < 0.0001, *I*^2^ = 0%), positive apprehension test (7.3% vs. 25.8%, *p* = 0.002, *I*^2^ = 11%), and subsequent surgical treatment for instability (5.6% vs. 37.8%, *p* < 0.00001, *I*^2^ = 0%) when compared with those underwent conservative management. And more patients returned to play (83.5% vs. 66.0%, *p* = 0.03, *I*^2^ = 81%) after arthroscopic Bankart repair. Outcomes regarding the functional scores did not reach a significant difference between the two cohorts.

**Conclusions:**

Arthroscopic Bankart repair showed superiority over conservative management in terms of recurrence, return to play, and subsequent instability surgery during the follow-up in young active patients that encountered first episode of dislocation. As long-term prognosis is comparable, an immediate surgical stabilization might not be suitable for everyone.

**Supplementary Information:**

The online version contains supplementary material available at 10.1186/s40001-023-01160-0.

## Background

Almost every orthopedic surgeon has to deal with anterior shoulder dislocation in their professional practices. Owing to its anatomical characteristics and increasing popularity of contact sports, shoulder is the most commonly dislocated joint and anterior shoulder dislocation accounts for nearly half of all joint dislocations [1]. A well functioned glenohumeral joint needs intactness and coordination of static component and dynamic force. Those structures that ensure normal function of shoulder are at high risks of injury at the moment of dislocation [2], including the glenoid labrum, bony glenoid rim, glenohumeral ligament, capsule, and humeral head.

Robinson et al. [3] reported an 87% to 100% incidence of Bankart lesion in first-time anterior dislocations. Eight nine percent incidence of Hill–Sachs lesions during first-time anterior shoulder dislocations was reported by Taylor et al. [4]. Nearly half of the dislocations occur in individuals aged 15 to 29, and the majority are males [5]. The recurrence of anterior shoulder instability could be as high as 87% in high-risk patients that managed conservatively after first episode of dislocation [6].

In a long-term prospective study, about half of the patients aged 20 to 25 who had encountered a primary anterior shoulder dislocation experienced recurrence of instability and about 25% needed surgical stabilization [7]. Follow-up data from the same group of patients revealed moderate to severe osteoarthritis in 18% of patients without recurrence. The corresponding figures were 26% for those undergoing surgical stabilization and 39% for patients with more than one recurrence who were managed conservatively [8]. The investigators had concluded that age older than 25 years at primary dislocation, recurrent instability, high-energy sports as the trigger of dislocation, and alcohol abuse were risk factors for developing osteoarthritis [8].

Patient expectation, age, gender, lifestyle, and sports level should all been taken into consideration during decision making process. There is a tendency towards arthroscopic stabilization at first episode of dislocation in recent years, especially for young patients with high-risk of recurrent instability. On the other hand, there was moderate-quality evidence that half of the patients did well after conservative management [9]. Previous systematic reviews have compared results of surgical and conservative treatment, with varied evidence levels and intervention methods [9–12]. New high-level evidence studies focusing on the comparison of arthroscopic Bankart repair (ABR) and the conservative treatment had emerged since then. In this context, we tried to pool available evidence and conduct an updated systematic review and meta-analysis on this topic.

## Methods

### Search strategy and data sources

We performed an electronic literature database search (Web of Science, Cochrane Library, Scopus, and Embase) in November 2021 by two independent investigators. The Boolean operators and search terms were as follows: (glenohumeral joint dislocation OR shoulder dislocation) AND (surgical OR operative OR repair OR arthroscopy OR arthroscopic) AND (immobilization OR nonsurgical OR nonoperative OR conservative), with no restriction on publication year or language. Bibliographies of included publications and previous relevant reviews were scrutinized to identify any additional studies that might be missed in electronic database search.

### Study selection and methodologic quality assessment

We followed the protocols established in the Preferred Reporting Items for Systematic Reviews and Meta-analyses (PRISMA) statement [13] (see Additional file 1). We included prospective cohort studies and randomized controlled trials comparing the effectiveness of arthroscopic and conservative management after first-time traumatic anterior shoulder dislocations. The mean follow-up duration should be at least 12 months. We excluded nonclinical studies (cadaveric, biomechanical, animal, or laboratory), and studies focusing on chronic shoulder instability, non-traumatic shoulder dislocation, or secondary shoulder instability. Methodological quality of included studies was assessed by two reviewers in accordance with a 12-item scale by the Cochrane Back Review Group [14], which consisted of assessing factors such as randomization, blinding, allocation concealment, selective reporting, and patient compliance. Disagreements were resolved by discussion.

### Data extraction

For each eligible study, two reviewers extracted relevant data independently with standardized tables and checked the accuracy. Specifically, we abstracted the study design, level of evidence, demographic data, intervention methods, follow-up duration, and loss to follow-up. Outcome measures of interest were recurrence of instability, return to play, subsequent instability surgery, and shoulder functional scores. We used the values from the original publications if available directly. Otherwise, we quantified eligible data presented only in figures or graphs with plot-digitizing software (Plot Digitizer, version 2.6.4; Joseph Huwaldt and Scott Steinhorst).

### Statistics

Statistical analysis was performed to compare the outcomes between two groups using Review Manager Software (Revman version 5.1.6., the Cochrane Collaboration, Oxford, United Kingdom). For the dichotomous data, relative risk (RR) was used, and for the continuous data, weighted mean difference (WMD) was used. Statistical heterogeneity was considered to be substantial when *I*^2^ (inconsistency) greater than 50%, and in this situation a random-effects model was used. To detect the impact of each study on overall results, a sensitivity analysis was conducted by sequentially deleting a single study involved. The level of significance was set at *p* < 0.05.

## Results

### Literature searching and patient demography

**Fig. 1:**
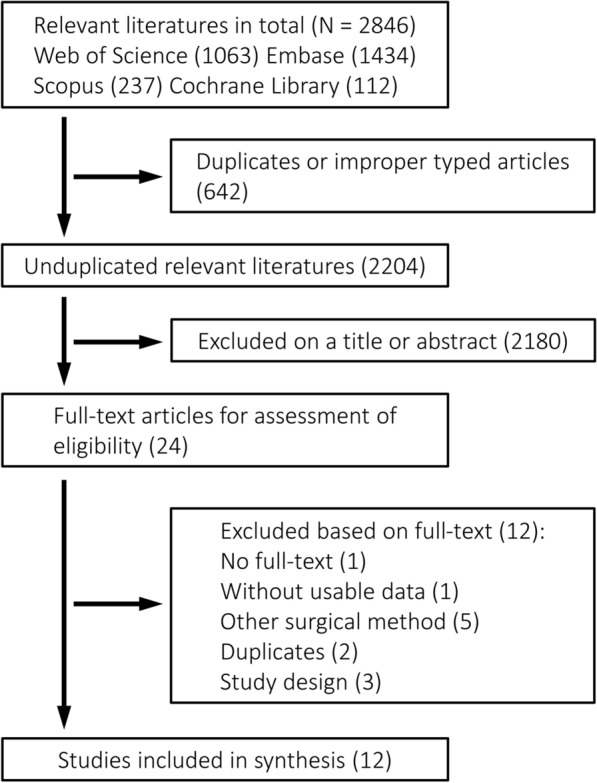
PRISMA (preferred reporting items for systematic reviews and meta-analyses) study selection flowchart

The literature search initially yielded 2846 relevant publications, of which 642 were excluded as duplicates. After title and abstract screening of the remaining, 24 potentially relevant studies were identified. By excluding 12 publications after full-text screening according to inclusion and exclusion criteria, 12 trials [3, 15–25] published from 1994 to 2021 with 786 participants were ultimately included (Fig. 1). Characteristics of included trials and demographic data are presented in Table 1. Male accounted for the majority of the population. Mean follow-up duration ranged from 12 to 93.8 months. The average age of the patients was 21.7 years and mean follow-up duration was 49.6 months. According to the 12-item standard of the Cochrane Back Review Group [14], 5 trials explicitly described randomization and 4 of them were rated as high quality (see Additional file 2).

**Table 1:** Overview of characteristics of included studies

Study	Regions	Year	Study design	Evidence level	Sample size	ABR vs. conservative	Age, year	Male/Female	Mean follow-up, month	Loss to follow-up (ABR vs. conservative)	Methodological quality
Pouge’s et al.	France	2021	RCT	I	40	20 ABR + IR 3wks vs. 20 IR 3 wks	21	33/7	24	0/0	High
Minkus et al.	Germany	2021	RCT	I	112	52 ABR + IR 3wks vs. 60 ER&ABD 3wks	26	103/9	24	8/13	High
De Carli et al.	Italy	2019	PCS	II	160	64 ABR + IR&ABD 4wks vs. 96 IR&ABD 4wks	21	121/9*	93.8	4/26	Moderate
Dickens et al.	USA	2017	PCS	II	39	29 ABR + ER&ABD 6wks vs. 10 without immobilization	20	36/3	12	0/0	Moderate
Gigis et al.	Germany	2014	PCS	II	72	43 ABR + IR 3wks vs. 29 IR 3wks	16	41/24*	36	5/2	Moderate
Shih et al.	Taiwan China	2011	PCS	II	67	39 ABR + immobilization 4 wks vs. 25 immobilization 4 wks	22	59/0^#^	71	3	Moderate
Robinson et al.	UK	2008	RCT	I	88	43 ABR + IR&ABD 6wks vs. 45 arthroscopic lavage only + IR&ABD 6wks	24	82/6	24	1/3	High
Kirkley et al.	Canada	2005	RCT	II	40	20 ABR + immobilization 3 wks vs. 20 immobilization 3 wks	23	35/5	79	4/5	High
Yanmis et al.	Turkey	2003	PCS	II	62	30 ABR vs. 32 immobilization	21	58/4	37	NA	Moderate
Bottoni et al.	USA	2002	RCT	I	24	10 ABR + immobilization 4 wks vs. 14 immobilization 4 wks	22	24/0	36	1/2	Moderate
Larrain et al.	Argentina	2001	PCS	II	46	28 ABR + immobilization 3–4 wks vs. 18 immobilization 2–4 wks	21	NA	67.4	0/0	Moderate
Arciero et al.	USA	1994	PCS	II	36	21 ABR + immobilization 4 wks vs. 15 immobilization 4 wks	20	NA	28.3	0/0	Moderate

RCT: Randomized Controlled Trial; PCS: Prospective Comparative Study; ABR: Arthroscopic Bankart Repair; IR: internal rotation; ER: external rotation; ABD: abduction; LR: Labral Repair;

*Analyzed patients; ^#^data originally presented by the study

### Recurrence and return to play

**Fig. 2:**
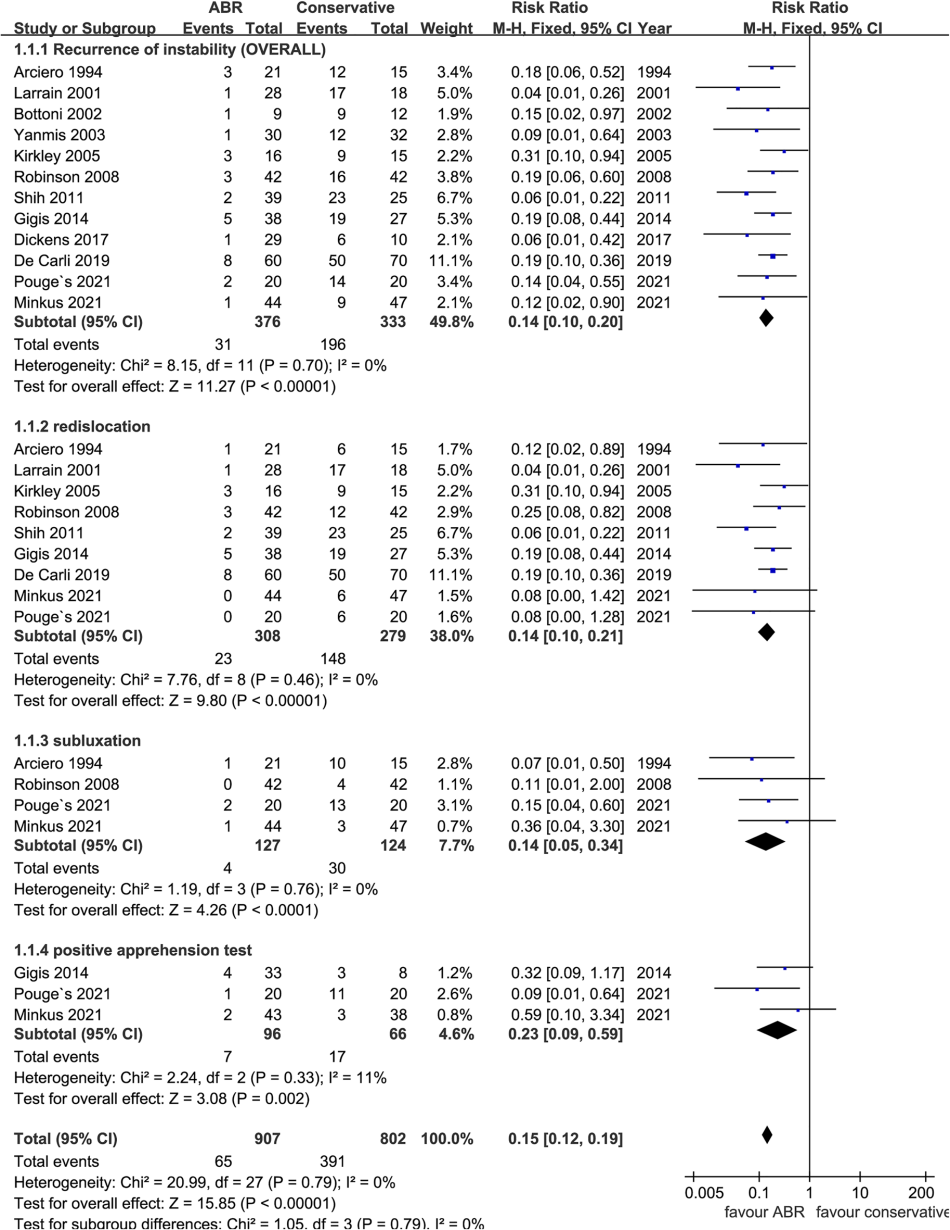
Forest plot of recurrent instability. ABR: arthroscopic Bankart repair; CI: confidence interval

All the included trials reported recurrent instability (overall, re-dislocation, subluxation, and/or positive apprehension test), and we collected the incidence of overall and/or specific instability events as detailed as possible. In summary, there were 31 patients (8.2%) in the ABR group experienced some form of recurrent shoulder instability postoperatively, while the number of conservative group was 196 (58.9%). The intergroup difference was statistically significant (*N* = 709, RR: 0.14 [0.10, 0.20]; *p* < 0.00001; *I*^2^ = 0%) and superiority of surgical management in regard of overall recurrent instability was obvious (Fig. 2). As for re-dislocation, 9 studies provided specific data and pooled results (*N* = 587, RR: 0.14 [0.10, 0.21]; *p* < 0.00001; *I*^2^ = 0%) demonstrated a significant lower re-dislocation rate in the ABR group (7.5%) when compared with conservative treatment (53.0%). Meanwhile, much fewer studies provided data on subluxation and positive apprehension test. According to results of four studies, only 3.1% of the patients (4 in 127) experienced subluxation after surgical stabilization when compared with 24.2% (30 in 124) of conservative group (*N* = 251, RR: 0.14 [0.05, 0.34]; *p* < 0.0001; *I*^2^ = 0%). And 7.3% of the patients (7 in 96) had positive apprehension tests after ABR when compared with 25.8% (17 in 66) of conservative group (*N* = 162, RR: 0.23 [0.09, 0.59]; *p* = 0.002; *I*^2^ = 11%).

**Fig. 3:**
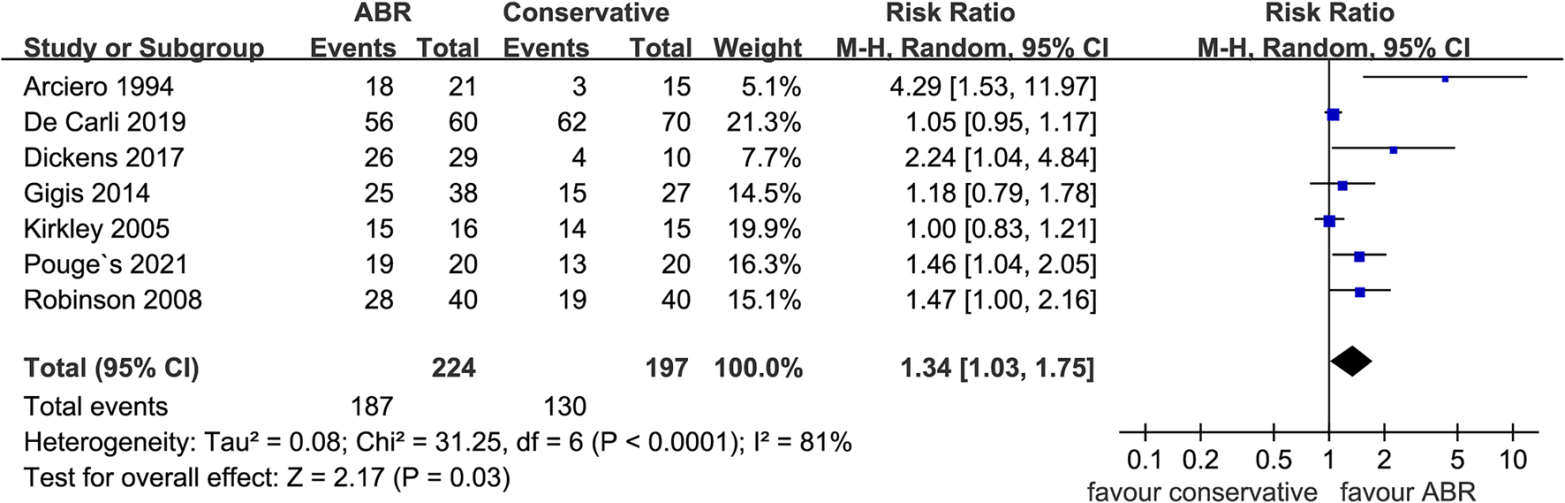
Forest plot of return to play. ABR: arthroscopic Bankart repair; CI: confidence interval

Return to play was reported in seven trials. It was an important measurement reflecting the effectiveness of two treatments as restoring the pre-dislocation level of sports activity was critical to young active patients. A statistically significant difference was noted in favor of ABR (*N* = 421, RR: 1.34 [1.03, 1.75]; *p* = 0.03; *I*^2^ = 81%) (Fig. 3) with 83.5% (187 in 224) of the patients return to play, while the data of conservative group was 66.0% (130 in 197).

### Subsequent instability surgery

**Fig. 4:**
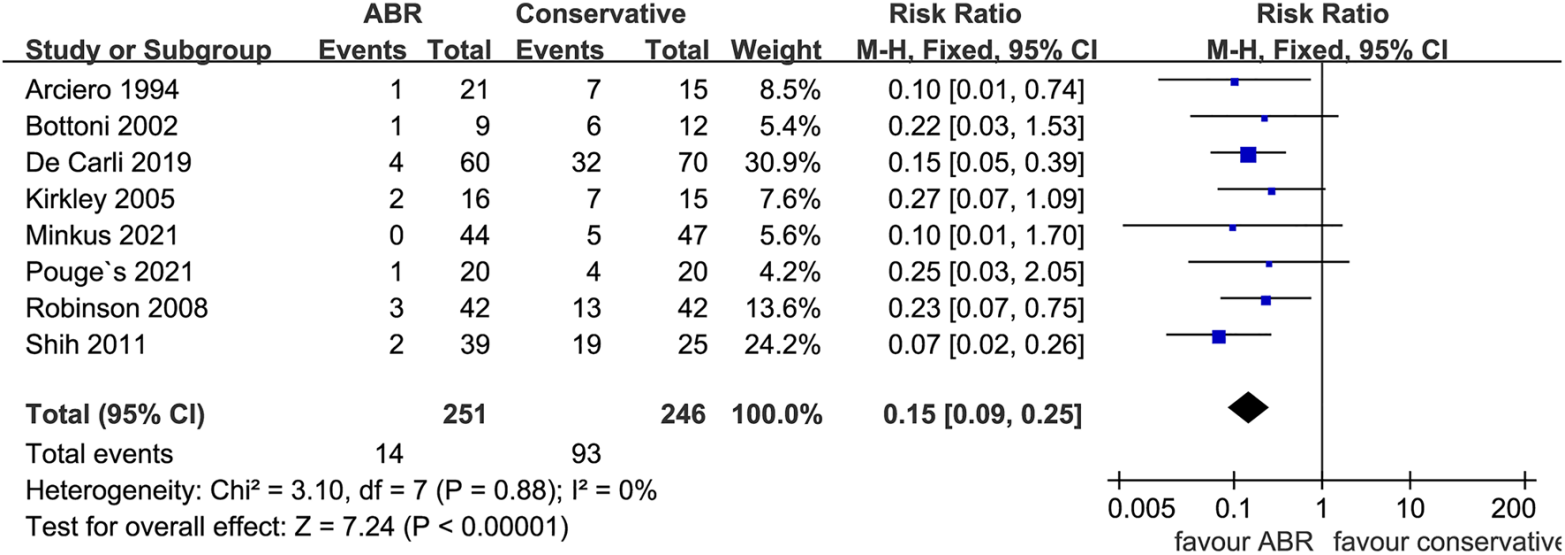
Forest plot of subsequent instability surgery. ABR: arthroscopic Bankart repair; CI: confidence interval

**Fig. 5:**
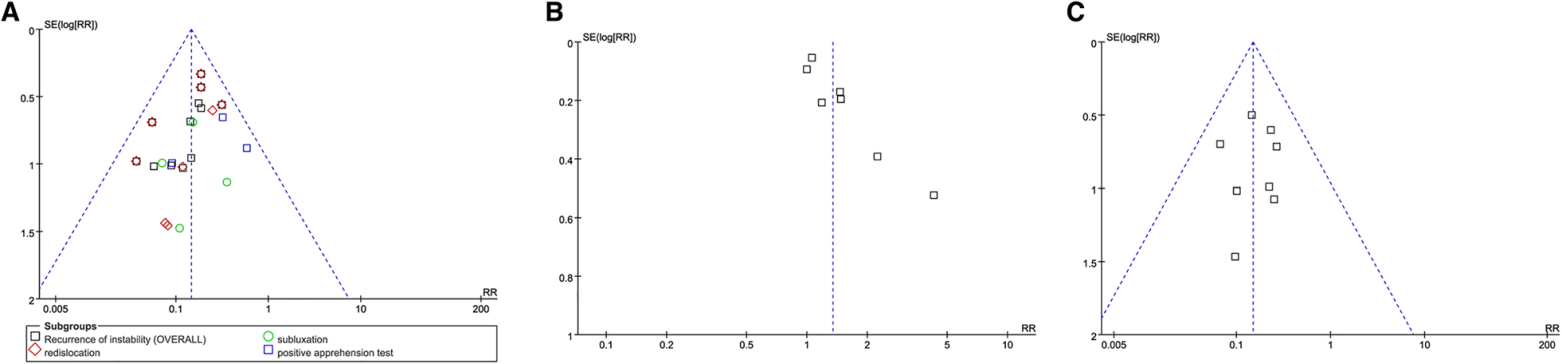
Funnel plots of recurrent instability (**A**), return to play (**B**), and subsequent instability surgery (**C**). Publication bias was detected as regard to the outcome of return to play. RR: relative risk

Eight studies provided data concerning subsequent instability surgery with 251 patients in ABR cohort and 246 patients in conservative cohort. Pooled results indicated 5.6% (14 in 251) of the ABR patients performed subsequent surgeries for shoulder instability, while the rate of conservative cohort was 37.8% (93 in 246). A statistically significant difference was detected in favor of ABR (*N* = 497, RR: 0.15 [0.09, 0.25]; *p* < 0.00001; *I*^2^ = 0%) (Fig. 4). And publication bias of the above mentioned major outcomes are present in Fig. 5.

### Functional scores

Functional evaluation was conducted in some studies and different scoring systems including Constant-Murley score (*N* = 153, WMD: 8.78 [− 14.64, 32.20]; *p* = 0.46; *I*^2^ = 98%), DASH score (*N* = 314, WMD: − 6.23 [− 14.56, 2.11]; *p* = 0.14; *I*^2^ = 98%), Rowe score (*N* = 262, WMD: 6.53 [− 7.96, 21.03]; *p* = 0.38; *I*^2^ = 98%), ASES score (*N* = 161, WMD: 5.86 [− 3.15, 14.88]; *p* = 0.20; *I*^2^ = 97%), and WOSI score (*N* = 372, WMD: − 4.57 [− 13.40, 4.26]; *p* = 0.31; *I*^2^ = 98%) were adopted. None of these reached a significant difference between the two cohorts.

## Discussion

Pooled results of prospective studies showed that arthroscopic stabilization significantly lowered the rate of recurrent instability after primary anterior shoulder dislocation in young active population. Evidence regarding functional scores failed to reveal a distinct difference between the two treatment arms due to variation and incompleteness of outcome measures among studies. As the study population were mostly young men and large part of the injury were sport related, the strategy of early surgical stabilization cannot be easily generalized to other groups of patients. For those with high expectations of returning to contact sports, ABR could be a reliable choice after the first episode of anterior dislocation. However, as long-term prognosis of instability and prevalence of dislocation arthropathy is comparable among ABR, nonoperative treatment, and open procedure [26, 27], a wait and see strategy or delaying surgery depending on recurrence may be a reasonable choice for the non-athletes.

Traumatic anterior shoulder dislocation is accompanied with high incidence of pathologic changes including osseous and soft tissue lesion. Bankart lesion (soft-tissue or bony avulsion) and Hill-Sachs lesion are the most common pathological findings in arthroscopic and radiological examinations. Glenoid labrum deepens the glenoid socket and increases its surface area, serving as a type of “chock block” to the humeral head [2]. And it’s an essential supplement to the glenohumeral stability besides the surrounding ligaments. Biomechanical study has revealed that both Bankart lesion and anterior glenohumeral ligament complex elongation contribute substantially to the occurrence of anterior glenohumeral instability [28]. Long term observation by Kavaja et al. indicated that mild glenohumeral arthropathy was common following conservative management [26]. However, results of ABR were comparable to conservative ones, which rarely caused more than minor subjective symptoms or objectively perceived disadvantages during long-term follow-up. Another 13-year radiological study also indicated that osteoarthritic changes were common findings after ABR, which were comparable to that after open repairs reported in the literature [27]. The energy of trauma and age of patient were deemed to be more relevant to recurrence and long-term arthropathy than the kind of treatment following shoulder dislocation [27, 29].

In terms of recurrent instability, the results revealed that ABR was superior as compared with conservative management in young active population, which is consistent with previous studies [10, 12, 29]. However, glenohumeral instability is encountered in a wide range of activities and age groups, making extrapolation to other groups of population like women, older patients, and non-athletes not necessarily feasible [9]. Results of a network meta-analysis [9] were less favorable towards surgery than other systematic reviews and meta-analysis [12, 30]. The authors attributed this difference to a strict inclusion of RCTs only, the use of network analysis, the handling of labrum repair, and arthroscopic lavage and non-surgical treatment as separate entities. According to a previous long-term follow-up study, about half of the first-time dislocation patients younger than 25 would experience recurrent instability and need stabilizing surgery, while another half had not recurred or become stable over time [7, 8, 31]. Also, observation of another group of patients indicated that delaying surgery depending on the development of chronic instability after first-time anterior traumatic shoulder dislocation did not necessarily lead to less favorable quality of life, inferior prognosis of instability or glenohumeral joint arthropathy [26]. Kavaja et al. held the opinion that a wait and see strategy would direct resources more efficiently than routine surgery after first-time anterior traumatic shoulder dislocation and might save half of patients from unnecessary surgery. In this context, the superiority of immediate arthroscopic stabilization in precaution of recurrent instability can be questioned. Even for young athletes who desired to return to athletic activity after first episode of dislocation, nonoperative management could be feasible and effective during the playing season [32]. And for those who experienced a dislocation and managed to complete the season, surgery can be delayed but not essential for everyone [32, 33].

Data of return to play also demonstrated a statistically significant difference between arthroscopic and conservative treatments after first-time traumatic anterior shoulder dislocations. As the majority of study population are young and active, returning to play seems to be a priority. But return to play not necessarily means no recurrence or successful treatment, as we noticed that varied standards were adopted among included studies. Some of them provided precise number of patients who returned to previous level of sports [21, 23], while some have no clear statement regarding whether the patients returned to activities of previous level or not [3, 22]. The same is true of the composition of participants, as only a small part of the studies [15–17, 22] enrolled pure athletes as research objects. The consistency among enrolled studies was not satisfactory due to above mentioned issues, and this led to a certain decrease of effectiveness of the statistically significant difference in the result of return to play. A uniform definition of return to play would have lend greater credence to the significance of our finding, and this need to be ameliorated in future studies.

We pooled available data regarding the function assessment. Final results concerning DASH score, (Western Ontario Shoulder Instability Index) WOSI score, Rowe score, ASES score, Constant-Murley score did not reach statically significant differences between groups. We also noticed that inconsistency (*I*^2^) of the results of functional scores among enrolled studies were too big to make an accurate comparison and solid conclusion. Additionally, these measurements include subjective items which might make it difficult to distinguish the superior treatment, especially when number of enrolled patients was relatively small.

Pooled result of subsequent instability surgery after primary anterior shoulder dislocation favored ABR without any suspense. The rate of recurrent instability was much higher after conservative treatment. It is reasonable that more additional surgery would be performed after primary treatment in that group. One major cause is that instability would cause inconvenient symptom in a quite portion of the patients and prevent them from participating in sports.

Functional evaluation failed to determine the better treatment. Number of studies that adopted functional evaluation as major measurement was relatively small. The reason we supposed was that dislocation caused varied trauma to the integrity of bony and soft structure, it might be difficult to apply a functional evaluation under various precondition.

Surgical timing is a crucial issue in handling shoulder instability. Duchman et al. found that glenohumeral bone and cartilage lesions were common findings at the time of both primary and revision shoulder stabilization surgery, and he appealed for further prospective study to compare the clinical results in these two groups [34]. Palth et al. pointed out that the age and extent of trauma sustained during preoperative dislocations were more relevant to long-term dislocation arthropathy than the kind of treatment [27]. Their study found that osteoarthritic changes at an average 13 years after ABR were comparable with that conservative treatment. Another radiologic evaluation and self-assessment study from Finland by Kavaja et al. indicated that incidence of glenohumeral arthrosis after ABR was quite common while the symptoms were generally mild and comparable to conservative treatment [26]. Though ABR can yield a more stable shoulder, these evidences indicated that long-term dislocation pathology was comparable between ABR and conservative treatment. Additionally, study by Barlow et al. failed to find a significant difference regarding recurrence rate in patients who had primary surgical stabilization after a single episode of dislocation compared with those experienced recurrent instability events [35]. And this further confirmed Plath’s view that the extent of trauma energy at primary dislocation was more relevant to recurrence [27]. It will be very interesting if more evidence on the relationship between traumatic energy and prognosis of shoulder dislocation could be disclosed in the future. More studies on long-term pathologic changes following both treatments should be present to provide more guidance for selecting the treatment.

The study has several limitations. Arthroscopic lavage was adopted in a conservative group in one study [3], whereas we pooled its result with other conservative ones. Previous studies [36, 37] revealed that arthroscopic lavage speeded reduction in effusion in the glenohumeral joint and lowered recurrence after primary anterior shoulder dislocation, however, its efficiency was deemed to be limited when compared with ABR. Sensitivity analysis also verified this. For post-reduction management, the immobilization position and duration were varied across the studies, and this is worthy of further unification in future studies. So is the definition of return to play, the unification of which would lend greater credence to the significance of pooled results. What’s more, adequate randomization was not achieved by most studies and this could reduce the reliability of the result. But so far, the pooled trials in this study presents the highest level of evidence available.

## Conclusions

When handling first-time traumatic anterior shoulder dislocation in young active population, ABR demonstrated superiority over conservative management in terms of recurrence, return to play, and subsequent instability surgery. However, it is necessary to distinguish non-athletes from athletes as long-term prognosis is comparable. An immediate surgical stabilization might not be a routine approach for everyone. Specific concern on patient anticipations, pathologic changes, future sport demands and gender should be addressed when making the decision of surgery.

## Supplementary Information

**Additional file 1.** PRISMA 2020 checklist. Identification of the location of the checklist item in the manuscript.

**Additional file 2.** Methodological quality of the included studies based on the 12-items scoring system. Statistical quality of included studies according to the 12-item standard of the Cochrane Back Review Group.

## Data Availability

All data generated during this study are included in this published article and its additional file. The original resources are the included studies of the review.

## Abbreviations

ABR: Arthroscopic Bankart repair
SLAP: Superior labral anteroposterior avulsion lesion
RR: Relative risk
WMD: Weighted mean difference
DASH: Disabilities of the Arm, Shoulder and Hand
WOSI: Western Ontario Shoulder Instability Index
RCT: Randomized controlled trial
ASES: American Shoulder and Elbow Surgeons
