# Supplementary material for: Arthroscopic Bankart repair versus conservative treatment for first-time traumatic anterior shoulder dislocation: a systematic review and meta-analysis
Source: Eur J Med Res. 2023 Jul 27;28:260. doi: 10.1186/s40001-023-01160-0 (PMC10373227; doi:10.1186/s40001-023-01160-0)
Supplement: Supplementary file 2 — Additional file 2. Methodological quality of the included studies based on the 12-items scoring system. Statistical quality of included studies according to the 12-item standard of the Cochrane Back Review Group. [file 40001_2023_1160_MOESM2_ESM.docx]

| Methodological quality of the included studies based on the 12-items scoring system | | | | | | | | | | | | | |
| --- | --- | --- | --- | --- | --- | --- | --- | --- | --- | --- | --- | --- | --- |
| Study | Randomized adequately^a^ | Allocation concealed | Similar baseline | Patient blinded | Care provider blinded | Outcome assessor blinded | Avoided selective reporting | Patient compliance^b^ | Similar or avoided cofactor | Acceptable drop-out rate^c^ | Similar timing | ITT analysis^d^ | Quality^e^ |
| Pouge`s et al. | Y | Y | Y | N | N | N | Y | Y | Y | Y | Y | Y | High |
| Minkus et al. | Y | Y | Y | N | N | N | Y | Y | Y | Y | Y | N | High |
| De Carli et al. | N | N | Y | N | N | N | Y | Y | Y | Y | Y | N | Moderate |
| Dickens et al. | N | N | Y | N | N | N | Y | N | Y | Y | Y | N | Moderate |
| Gigis et al. | N | N | Y | N | N | N | Y | Y | Y | Y | Y | N | Moderate |
| Shih et al. | N | N | Y | N | N | N | Y | Y | Y | Y | Y | N | Moderate |
| Robinson et al. | Y | Y | Y | Y | Y | N | Y | Y | Y | Y | Y | Y | High |
| Kirkley et al. | Y | Y | Y | N | N | Y | Y | Y | Y | N | Y | Y | High |
| Yanmis et al. | N | N | Y | N | N | N | Y | Y | Y | N | Y | N | Moderate |
| Bottoni et al. | Y | N | Y | N | N | N | Y | Y | Y | Y | Y | N | Moderate |
| Larrain et al. | N | N | Y | N | N | N | Y | Y | Y | Y | Y | N | Moderate |
| Arciero et al. | N | N | Y | N | N | N | Y | Y | Y | Y | Y | N | Moderate |
| ^a^ Only if the method of sequence generated was explicitly described could got a "Yes"; otherwise received a "No". | | | | | | | | | | | | | |
| ^b^ Mean follow-up duration >/= 24 months means "Yes", otherwise "No". | | | | | | | | | | | | | |
| ^c^ Drop-out rate </= 20% means "Yes", otherwise "No". | | | | | | | | | | | | | |
| ^d^ ITT = intention-to-treat, only if all randomised patients are analysed in the group they were allocated to could receive a "Yes", otherwise "No". | | | | | | | | | | | | | |
| ^e^ The frequences of "Yes" >/= 8 means "High"; >/= 4 and < 8 means "Moderate"; < 4 means "Low". | | | | | | | | | | | | | |
